# Risk Prediction of Emergency Department Revisit 30 Days Post Discharge: A Prospective Study

**DOI:** 10.1371/journal.pone.0112944

**Published:** 2014-11-13

**Authors:** Shiying Hao, Bo Jin, Andrew Young Shin, Yifan Zhao, Chunqing Zhu, Zhen Li, Zhongkai Hu, Changlin Fu, Jun Ji, Yong Wang, Yingzhen Zhao, Dorothy Dai, Devore S. Culver, Shaun T. Alfreds, Todd Rogow, Frank Stearns, Karl G. Sylvester, Eric Widen, Xuefeng B. Ling

**Affiliations:** 1 HBI Solutions Inc., Palo Alto, California, United States of America; 2 Department of Surgery, Stanford University, Stanford, California, United States of America; 3 Department of Pediatrics, Stanford University, Stanford, California, United States of America; 4 Department of Statistics, Stanford University, Stanford, California, United States of America; 5 HealthInfoNet, Portland, Maine, United States of America; 6 Academy of Mathematics and Systems Science, Chinese Academy of Sciences, Beijing, China; University of Catania, Italy

## Abstract

**Background:**

Among patients who are discharged from the Emergency Department (ED), about 3% return within 30 days. Revisits can be related to the nature of the disease, medical errors, and/or inadequate diagnoses and treatment during their initial ED visit. Identification of high-risk patient population can help device new strategies for improved ED care with reduced ED utilization.

**Methods and Findings:**

A decision tree based model with discriminant Electronic Medical Record (EMR) features was developed and validated, estimating patient ED 30 day revisit risk. A retrospective cohort of 293,461 ED encounters from HealthInfoNet (HIN), Maine's Health Information Exchange (HIE), between January 1, 2012 and December 31, 2012, was assembled with the associated patients' demographic information and one-year clinical histories before the discharge date as the inputs. To validate, a prospective cohort of 193,886 encounters between January 1, 2013 and June 30, 2013 was constructed. The *c*-statistics for the retrospective and prospective predictions were 0.710 and 0.704 respectively. Clinical resource utilization, including ED use, was analyzed as a function of the ED risk score. Cluster analysis of high-risk patients identified discrete sub-populations with distinctive demographic, clinical and resource utilization patterns.

**Conclusions:**

Our ED 30-day revisit model was prospectively validated on the Maine State HIN secure statewide data system. Future integration of our ED predictive analytics into the ED care work flow may lead to increased opportunities for targeted care intervention to reduce ED resource burden and overall healthcare expense, and improve outcomes.

## Introduction

The rapid growth of the emergency department (ED) visits in last few years in US demands larger healthcare resources than ever [1]. Between 2001 and 2008, the annual number of US ED visits grew at roughly twice the rate of population increase [2]. Among the high volume of the ED visits every year, ED return rates are considerable. More than 50% of Massachusetts residents endured multiple visits, and that 1% had 5 or more ED visits which construct 18% of all visits in the state [3]. 8% of Veterans Health Administration (VHA) patients had ED revisits in 2010, almost equal to those who had single ED visit in the same year [4]. The national prevalent health delivery problem [5] of over-crowded EDs has imposed a highly consistent day-to-day burden on hospital resource utilization [6], driving the US EDs to a breaking point as described by the Institute of Medicine [7]. The vulnerable population to ED return is therefore of public interest, especially with regard to healthcare beneficiaries concerned with decreasing morbidity and costs [8], and has encouraged the US government in efforts to prevent avoidable ED mis-use or reuse.

Earlier studies focusing on ED revisits revealed that there are various driving factors for those post-discharge returns, including nature of the disease, medical errors [9], patient satisfactions [10], and inadequacy of initial evaluation or treatment [11]. Frequent returns can also be caused by over-estimation of the medical situations unnecessary for ED revisit [12], [13]. Investigations also demonstrated that ED returns occurring shortly after discharge were mainly unscheduled [14] that were highly correlated to diagnostic errors and insufficient care or follow-up [15], indicating that those revisits may serve as an important target for quality assurance of the medical care. Presuming a large proportion of unexpected short ED return visits are avoidable by more knowledgeable patients or more definitive diagnoses or procedures at the initial visits [16], predictive analytics of ED 30 day revisit may device appropriate strategy of discharge planning and ED utilization, improving quality of patients' care and controlling healthcare expenditures [17].

Accurate prediction of ED return visits is an integral component to assist cost-effective resource allocation planning seeking to improve post discharge intervention in high-risk patients. Early efforts on this topic included risk prediction models for hospital readmission [18] and repeated ED visit for patients with distinct patterns [19]–[21]. Although previous studies have demonstrated limited utility of certain settings [22] and identified risk factors for the ED return [23], [24], little is known about the ED revisit risk prediction, especially on the revisits in the same reasons occurring shortly after discharge [16], [25]. Furthermore, currently used prediction models have limitations. They either rely on data systems biased by the high rate of previous ED admissions that do not necessarily correlate with ongoing risk for future ED admission [26], or focus on patients within specific payer groups [27], e.g. Medicare, within specific age [28], [29], and/or within specific disease groups [30], [31]. Many of the studies on ED return prediction only reported their analysis by p-values and odds ratios [14], [32]–[35], or were not validated prospectively [10], [36]. A systematic review stated that many readmission prediction models currently available didn't have sufficiently high performances for clinical use [18]. Efforts are needed to develop more comprehensive ED revisit risk methods, which allow prospective identification of various levels of ED return risk subjects from heterogeneous ED population.

The development of EMR systems and health information exchanges (HIE) in US makes clinical information available covering a broad scope of patients of all payers, all ages, and all diseases, inciting more comprehensive studies on healthcare services utilizing the patients' comprehensive characteristics. In this study, we set to develop a predictive model from patient information contained in the statewide HIE of longitudinal patterns to estimate the probability of a ED revisit in future 30 days after discharge. Our study is one of the first of its kind to study and predict statewide ED revisit risk in 30 days across all payers, all diseases and all age groups.

## Methods

### Ethics statements

This work was done under a business/product development arrangement between HIN and HBI Solutions, Inc. and the data use is governed by the business agreement (BAA) between HIN and HBI. No PHI was released for the purpose of research. Instead, HBI completed the product development that was the foundation for our agreement and then reported on the findings resulting from applying this model to the products/services that HIN is now deploying in the field.

### Population

The study targeted to cover patients visiting any HIN connected facility from January 1, 2012 through June 30, 2013, with the following exclusions: (1) patients that died during the study time frame of 2012 and 2013; (2) patients that did not have any primary diagnoses, partly due to the HIE removal of mental health or substance abuse diagnoses as mandated by Maine State law. ED visits that transferred from another ED were treated as single ED visit. All the ED visits included in this study were “unplanned”.

### Data warehouse

We constructed an enterprise data warehouse consisting of all Maine's HIE aggregated patient histories. The Maine HIE went live in 2009 and now contains records for close to all of Maine residents and is connected to the majority of health care facilities in Maine. There are currently 475 facilities connected to the Maine HIE including 376 physician offices, 12 behavioral health facilities, 15 critical access hospitals, 37 federally qualified health centers (FQHC), 23 hospitals, and 12 long-term care facilities. The HIE includes records for 1.35 million individuals including in-state and out-of-state residents. Over 90% of Maine residents have a record in the database. HealthInfoNet is an independent, nonprofit organization operating the HIE in Maine. It maintains an opt-out consent process for general medical information and an opt-in patient consent for certain behavioral health and HIV related information as required by Maine State law. The HIE has just over a 1% patient opt-out rate. Incorporated data elements from EMR encounters include patient demographic information, laboratory tests and results, radiographic procedures, medication prescriptions, diagnosis and procedures which are coded according to the *International Classification of Diseases, 9^th^ Revision, Clinical Modification* (ICD-9-CM). Census data from the U.S. Department of Commerce Census Bureau were integrated into our data warehouse, to provide approximation on patients' socioeconomic status information in terms of the average household mean and median family income and average degree of educational attainment, based on residence zip codes.

There are totally 14,680 features describing the profile of patient clinical history, with many of zeros for each. Feature selection according to the data variance [37] was exploited before modeling process to reduce the redundancy. As a result, 127 features in the prior 12 months to the ED discharge date were selected as inputs for the subsequent modeling (Table S1). One of the key features was whether the patient had a chronic medical condition. This feature was defined using the AHRQ Chronic Condition Indicator [38] (CCI) which provides an effective way to categorize ICD-9-CM diagnosis codes into one of two categories: chronic and non-chronic.

### Overview of study design

**Figure 1:**
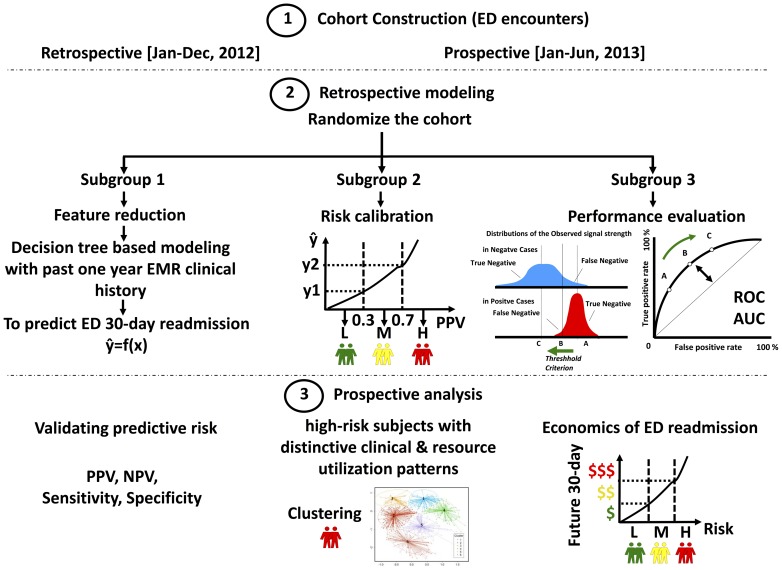
Study design to develop the ED 30 day revisit predictive algorithm. There were three main steps for model development: 1) two independent cohorts were constructed for retrospective modelling and prospective validation; 2) samples in the retrospective cohort were used to train a decision-tree-based predictive model, followed by a calibration and blind-test procedure; 3) the model integrating a risk-score metric was validated on the prospective cohort for further performance analysis.

The statistical learning to forecast future 30-day ED revisit risk consisted of two phases: retrospective modeling and prospective validation (Figure 1).

### Cohort construction

**Figure 2:**
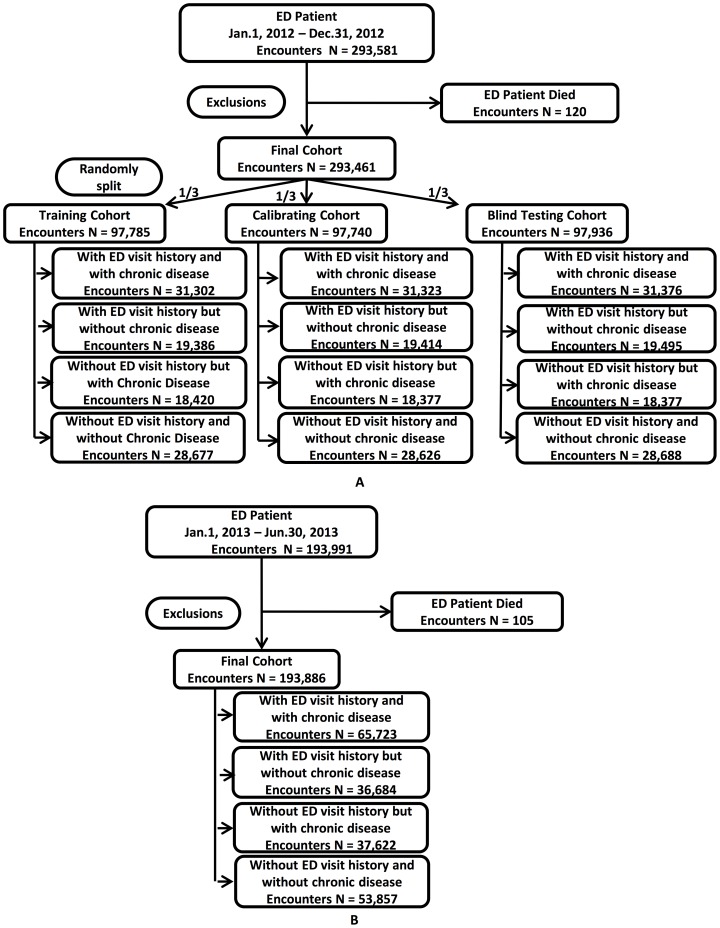
Study cohort construction (A, retrospective; B, prospective), and inclusion/exclusion criteria.

A retrospective cohort of 293,461 ED encounters (Figure 2A), between January 1, 2012 and December 31, 2012, was assembled to develop the model to predict ED revisit within 30 days post discharge. The model was validated by a prospective cohort of 193,886 encounters (Figure 2B) between January 1, 2013 and June 30, 2013. Both cohorts associated patients had similar demographics and one-year comprehensive clinical histories before the discharged date. Prior year EMR data before the ED discharge was used to allow the determination of post discharge ED revisit risk (Table S2).

The study targeted the population in Maine. Within a given period, the total population remains approximately the same, with minor immigration and emigration. However, given that the analysis in this study was event-based (ED-visit-based), there were no overlap between the retrospective and prospective cohorts.

### Exploratory data analysis

Unscheduled ED revisits may occur for any reason and can be separated by days, weeks, months or years. ED revisits could be due to the received poor quality or for unexpected complications. When selecting an appropriate time period for the revisit, we considered selecting a time interval that allows for the same risk of exposure of all patients as a population, within which the revisits tended to raise healthcare utilization issues.

**Figure 3:**
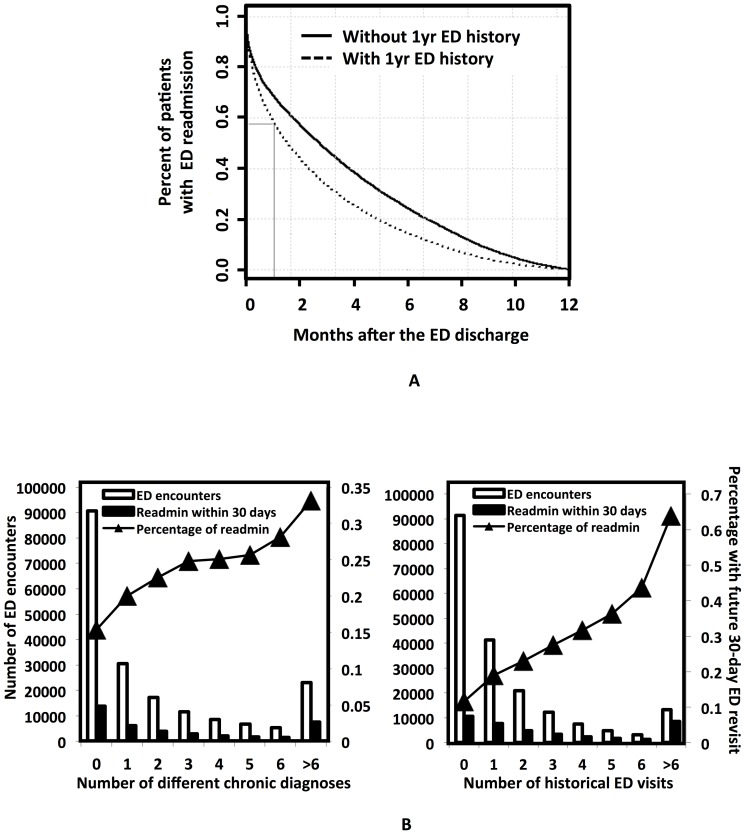
Exploratory data analysis. A. “Time to event” analysis. The ED revisit “time-to-event curve” showed a pattern of a rapid accrual with a stable and consistent ED visit rate thereafter. The population ED revisit curves, of patients with or without past history of ED visits, decreased significantly within 30 days from the ED discharge time, indicating that a 30-day cutoff is clinically reasonable. B. Our analysis found that both the total number and the percentage of patients with future 30-day ED visits increased as a functional of either the distinct chronic diagnoses (left panel) or the ED visit counts (right) in the prior 12 months.

Prior to the model development, we reviewed a “time to event” curve of ED revisits of the retrospective cohort to determine whether 30-day post discharge ED revisit assessment is clinically reasonable. The ED revisit “time-to-event curve” (Figure 3A, Figure S1) showed a pattern of rapid accrual with a stable and consistent ED revisit rate thereafter. The percentage of patients having no ED revisit within 30 days after discharge reduced to less than 60% from the discharge time for those having ED history and 70% for those without ED history, clearly imposing a burden on hospital resource utilization. It indicated that a 30-day cutoff is reasonable and appropriate for this study. Similar incidence of future 30-day ED revisits in retrospective and prospective cohorts (retrospective: 19.4%; prospective: 20.5%; Table S2) indicated the model developed retrospectively can possibly be used to describe the prospective behaviors. Our exploratory analysis (Figure 3B) of the retrospective cohort showed that the percentage of ED revisits increased as a function of either historic ED visit counts or the presence of chronic disease diagnoses, therefore, these two features were strongly associated with patients' risk for ED revisits.

### Model development – A retrospective analysis

The goal of the present study was to develop an ED revisit prediction algorithm to measure a statewide post discharge 30-day ED revisit risk. Decision trees were constructed during the model development to generate scores estimating the probability of the revisit upon one year of the encounter history. The retrospective modeling phase consisted of three steps: (1) training, (2) calibrating, and (3) blind testing. As indicated in Figure 2A, the samples in the retrospective cohort were divided into four subgroups based on histories of chronic diseases and ED visits. Then, in each subgroup, the retrospective cohort case (post discharge 30-day ED revisit counts>0) and control (post discharge 30-day ED revisit counts  = 0) samples were randomly split into training, calibrating and blind-testing cohorts (Figure 1), with consideration that the past 12-month ED histories of encounters achieved a balance. i.e. The ED visits across the past 12 months were averagely distributed on a monthly basis among all 3 cohorts.

#### Modeling Step (I)

A “survival forest” of forecasting decision trees was developed using the prior year clinical history, and ranked according to the corresponding posterior probability. Specifically, a ‘*Tree*’ model was developed using the prior year clinical history (‘*Data*’), First, a general technique of bootstrap aggregating (bagging) [39] was applied to randomly bootstrap sample of the entire training cohort for growing the tree; second, the survival trees were grown based on the randomly selected predictors via log-rank survival splitting rule on each survival tree node [40]. 
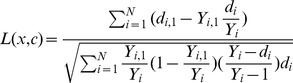



Here, *c* is the split value for predictor *x*, *d_i,_*_j_ and *Y_i,j_* for node *h* equal the number of patients who has ED return event in *t_i_* day after discharge and who never come back in *t_i_* day after discharge for daughter nodes *j* = 1, 2. Hence, *Y_i,1_  =  |*{*T_l_> =  t_i_* & *x_l_ < =  c*}*|* and *Y_i,2_  =  |*{*T_l_> =  t_i_ & x_l_*>*c*}|, where *T_l_* is the days for patient come back to ED after discharge for the individual *l*. The value *|L(x, c)|* is the measure of node separation, which quantifies splitting for the predictor x when split value equal c. Therefore, the optimized predictor x^*^ and split value c^*^ at node *h* is determined by maximizing the |L(x^*^, c^*^)|> =  |L(x, c)| for all *x* and *c*.

Third, an ensemble cumulative hazard estimate by combining information from the survival trees so that each individual will be assigned one estimate. 
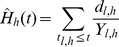



Where 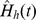 is the cumulative hazard estimate for node *h*, *t_l,h_* is the distinct death times in node *h*, *d_l,h_* and *Y_l,h_* represent the number of deaths and individuals at risk at time *t_l,h_*. 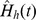 was computed for terminal node for each predictor *x_i_* for individual sample *i* drop down into in the tree. We implemented ntree  = 300 to grow the “survival forest”, and ensemble the cumulative hazard estimate for each tree together within the forest to calculate final predictive scores for each individual patient. Therefore, 
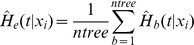



Here *b* denotes the individual tree and *ntree* is the number of trees in survival forest.

#### Modeling Step (II)

Cohort II was used to calibrate the predictive scores calculated from Step (I) by creating a risk measure for each score. Applying the Step (I) model to each sample *i* in Cohort II, the derived predictive scores 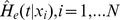 were ranked.

For each value of T, we can calculate the positive predictive value (PPV) as follow: 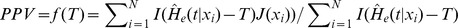 where
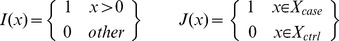
and *X_case_* and *X_ctrl_* denote the patients who have ED revisit and never have ED visit in 30 days after discharge.

In this way we have a mathematic function mapping predictive values to PPVs. i.e. each sample *i* was assigned a PPV to estimate the risk of becoming a case (having ED revisit in 30 days) with the given score. The PPV values were converted to a value ranging from 0–100 to define a risk level. For example, a sample had a predicted value associated with PPV index of 80 meant this sample had 80% probability to make ED return in 30 days. Its risk level is 80.

We obtained two thresholds *T_h_*, *T_m_* from this mapping.
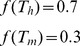



Then we stratified the patients into three risk groups

High risk group:
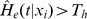



Intermediate risk group:
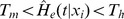



Low risk group: 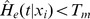



#### Feature selection

**Figure 4:**
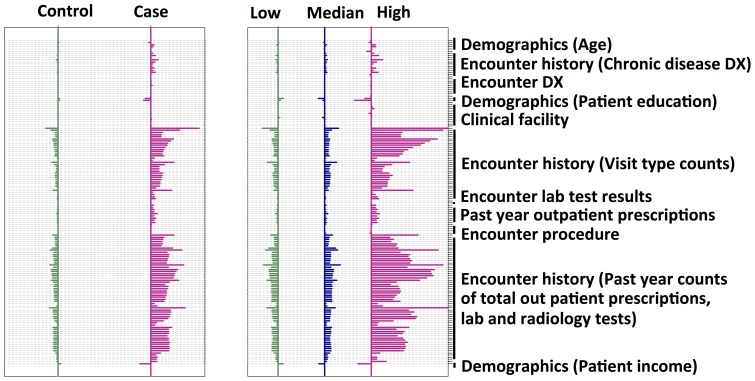
Characterization of the discriminant features in the prospective data set. Shrunken difference for the selected features to develop the ED risk model were graphed in order to measure the feature abilities in discriminating different classes. The x axis is the shrunken difference of each feature listed along the y axis, which is a measure of the difference between the standardized mean value of a feature within a specific class and the overall mean value of that feature. Comparing the two cohorts (case/control or the low/medium/high risk), the shrunken differences of these discriminative features were much more pronounced in the low/medium/high risk cohort, demonstrating the effectiveness of these features in prospectively differentiating the targeted outcomes.

To identify the discriminant features and avoid under and/or over fitting during the statistical learning, we applied a feature selection process (Figure S2). 2000 features were first selected from the 14,680 features, by choosing the top 2000 features of sufficient variation. Then a random forest model was built based on these 2000 features. A list of the features and importance was generated from the random forest model. A second round modeling was thereafter done by using the stop 10, 20, 30, 40, 50, 60, 70, 80, 90, 100 features from the feature list. A best ensemble model was chosen according to the performance of sensitivity, specificity and PPV (Figure S3). As a result, 127 variables predictive of future 30-day risk of ED visit were identified: demographics groups (9), different encounter history (84), care facilities (10), primary and secondary diagnoses (8), primary and secondary procedures (1), chronic disease condition (8), laboratory test results (2), and outpatient prescription medications (5). These features' shrunken difference [41] (Prospective analysis: Figure 4) were grouped according to the risk level categories identified above. These discriminant features' absolute values of the shrunken differences, among the low, medium, and high risk outcomes, differed more than the case (with future ED) and control (without future ED) outcomes, prospectively demonstrating the effectiveness of these features in the risk stratification.

#### Modeling Step (III)

After calibration, the model's performance was blind tested by Cohort III, with purpose of assessing the model and calibration values derived in Step (I) and (II). Again we applied the Step (I) model to each sample *i* in Cohort III to derive the predictive scores 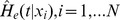 and worked out the risk levels according the PPV-score mapping constructed in Step (II). The AUC score for Cohort III was also calculated as described in Step (II) analysis. The derived predictive scores 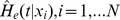 were ranked, and the AUC score was computed as following:
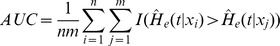



### Model validation – A prospective analysis

The derived ED 30-day revisit risk estimation algorithm was validated using an independent cohort with prospective HIE data in Maine in order to explore its statewide application. The receiver operating characteristics (ROC) [42] and time to event analyses were performed and compared to those of retrospective tests to gauge the model performance and effectiveness of the risk stratification.

### Use of ED scoring metric to forecast the economic impact of ED revisits

Use of the ED revisit risk scoring metric to forecast future ED and other resource utilization would indicate the clinical utility of our risk metric. Each encounter-based cost was computed, and each subject's future cost values were estimated based on a combination of encounter types (surgical/medical outpatient, ED visit, and inpatient), diagnosis, and procedure CCS group [43], [44]. 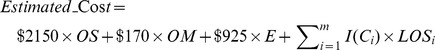
where *OS*, *OM*, *E* are the surgical outpatient, medical outpatient, emergency visit counts respectively in future 30 days after discharge, *LOS_i_* is inpatient length of day for *i_th_* inpatient encounter within 30 day after discharge, and *I(C_i_)* is the cost map function presenting the cost per day for specific inpatient diagnosis, and procedure category *C_i_*.

The resource utilization of all different encounters or ED encounters for each patient, post ED discharge future 30 days, was summarized at different risk levels defined by our model.

### Unsupervised clustering of high risk ED patients to reveal distinctive sub-populations for targeted care

To reduce high dimensional EMR features for detecting cohort pattern, we used principle component analysis (PCA) [45] to divide the high risk patients of 30-day ED return identified by our algorithm in the prospective cohort into distinctive groups, based on demographics, primary diagnosis and procedure, and chronic disease conditions. The features for high-risk patients are projected to a lower dimensional subspace with largest variances: 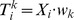



Where *X_i_* is EMR feature matrix for each high-risk patient, and *w_k_* is the set of vectors of weights that map each patient feature vector *X_i_* to a new vector of principal component scores *T_i_^k^*. And we computed *w_1_* by solving following objective functions (1) and (2) and *w_k_* by iterating objective function (3) based on the first *k*-1 principal components,
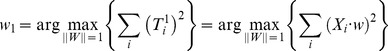


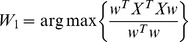


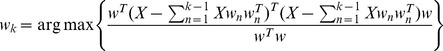



And then K-means algorithm was applied on the top of principal components *T_i_^k^* subspace of PCA to find potential patient patterns for 30-day ED return [46]. We used *K* = 6 to implement initial k means set for the algorithm and calculate the *Euclidean* centroid *m* to generate finial clusters,
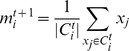



Where *C_i_* is the *i_th_* cluster in total 6 clusters, and *x* represents the previous principal components *T^k^*.

Unique patterns revealed by the clustering results were analyzed to characterize the high-risk subjects identified by our ED algorithm.

## Results

**Figure 5:**
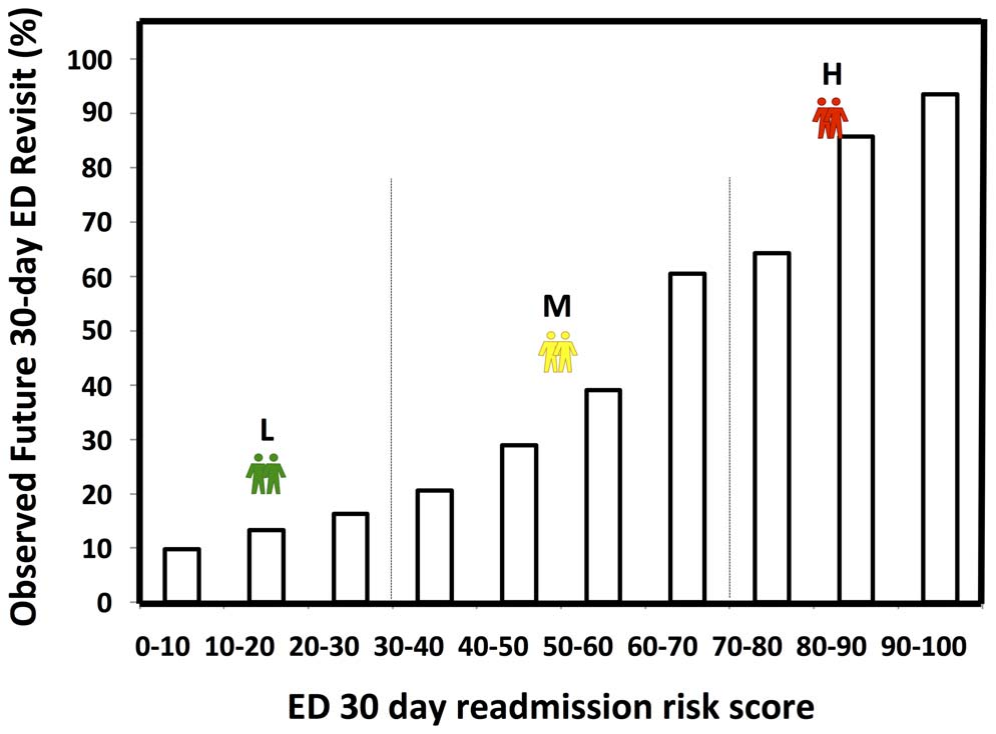
Observed rates of future 30-day ED returns versus risk scores in prospective tests.

Our ED revisit algorithm produced a risk score (from 0 to 100) for each patient at ED discharge to assess the risk of ED revisit. The trending of PPVs and sensitivities as a function of risk scores were similar in both retrospective with prospective analyses, indicating the robustness of the model (Figure 5, Table S3). The PPV values increased monotonically as the risk scores went high. When the risk score was more than 60, the model identified more than 60% of the ED 30 day revisits in prospective tests. With a risk score higher than 90, 93.5% of prospective revisits were identified correctly. At risk scores between 30 and 40 in prospective analysis, the algorithm found a fairly impressive percentage (24.4%) of all ED revisits. Sensitivities decreased with the risk increase, up to 3.0% with scores higher than 70. The receiver operating characteristic curve analyses showed that there was a 71.0% (retrospective) or 70.4% (prospective) probability that a randomly selected ED discharged patient with a 30-day post discharge ED revisit will receive a higher risk score than a randomly selected patient who will not have a future 30-day ED revisit.

**Figure 6:**
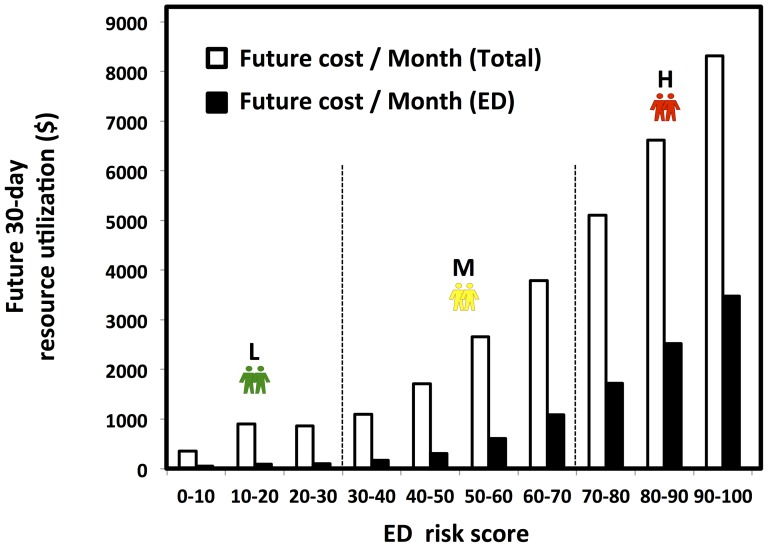
Future 30-day resource utilization analysis as a function of the ED risks. PMP1M: per member per 1 month. Two vertical lines serves as the boundaries between low, medium, high ED revisit risks.

**Figure 7:**
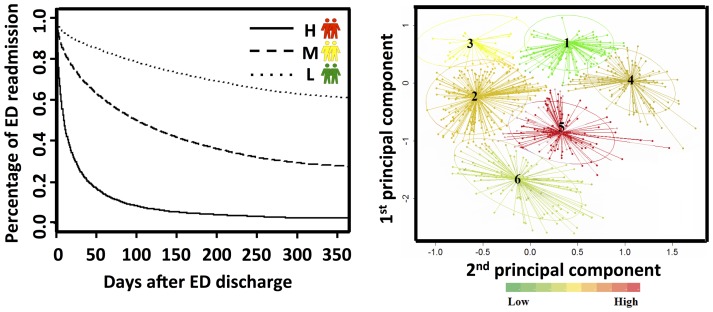
The ED predictive algorithm effectively risk-stratified the prospective patient cohort for future 30-day ED visit. Left panel: “Time to event” graphic representation of the low, medium and high risk patients' time to the next impending ED visit. Right panel: Unsupervised clustering of the high-risk patients identified distinct subgroups in the prospective cohort. Color-coding reflects the average cost of the high-risk patients in the next 30-day post discharge.

In addition to reasonable PPV and sensitivity values, our prospective analysis illustrated that there are distinct level changes in resource utilization from the low risk group to high risk group, revealing that our ED revisit risk can be used to forecast the trending of both the total patient care expense and ED resource utilization post ED discharge 30 days (Figure 6). Patients in higher risk categories returned to the ED earlier (prospective time to event analysis: *p*<0.001, Figure 7, left panel) and more frequently (Table 1) over the post discharge 30-day period.

**Table 1:** Clustering of ED-30 day high risk patients in the prospective cohort.

Characteristics	Cluster					
	1	2	3	4	5	6
**Utilities**						
Number of Encounters	842	773	641	387	376	365
Average total lab test counts	351.08	502.33	308.29	607.38	1143.30	542.09
Average total Radiology counts	8.23	15.60	8.21	9.87	56.45	21.31
Average future 30-day ED counts	1.41	2.16	2.23	2.48	1.86	2.13
Average future 30-day cost ($)	3641.77	5454.20	5046.48	5405.67	8244.95	4751.95
Total future 30-day cost ($) per cluster ×10^6^	3.07	4.22	3.24	2.09	3.10	1.74
**Demographics**						
Sex (Female)	64.37	29.88	20.28	89.66	38.30	81.10
Age new born	0.00	0.00	0.31	0.26	0.00	0.00
Age 1–5	0.24	0.13	1.87	0.00	0.00	0.00
Age 5–12	0.12	0.00	0.47	0.00	0.00	0.00
Age 12–18	0.36	0.00	3.90	1.03	0.00	0.27
Age 18–35	93.71	1.55	20.59	98.45	0.00	15.89
Age 35–50	2.49	59.12	49.61	0.26	38.56	41.37
Age 50–65	0.36	30.53	19.34	0.00	48.40	36.44
Age>65	2.73	8.67	3.90	0.00	13.03	6.03
**Chronic disease DX**						
Total chronic disease DX	2.05	4.90	0.56	6.33	15.36	9.50
Percentage of encounters without chronic diseases	13.90	0.00	70.98	0.00	0.00	0.00
Other nervous system disorders	21.97	47.09	0.00	56.33	88.03	75.34
Essential hypertension	8.31	55.11	0.78	14.73	90.69	48.77
Esophageal disorders	10.69	11.38	0.78	58.14	82.18	73.42
Asthma	16.15	12.55	0.94	74.42	49.20	58.36
Other nutritional; endocrine; and metabolic disorders	4.51	12.55	0.00	41.60	61.97	61.64
Disorders of lipid metabolism	1.19	19.79	0.00	7.24	84.57	33.15
Diabetes mellitus without complication	1.19	20.57	2.18	15.50	61.70	39.18
Chronic obstructive pulmonary disease and bronchiectasis	0.12	26.78	0.16	2.33	66.22	32.60
Headache; including migraine	9.74	5.56	1.72	27.39	8.78	33.15
Epilepsy; convulsions	4.39	11.90	2.96	21.96	21.28	18.63
Diabetes mellitus with complications	1.07	10.61	0.62	2.58	57.18	15.07
Thyroid disorders	4.16	5.05	1.56	16.54	31.65	20.82
Spondylosis; intervertebral disc disorders; other back problems	3.92	9.44	0.31	9.30	30.32	18.08
Cardiac dysrhythmias	3.09	10.09	0.78	10.59	31.38	13.15
**Chronic disease DX**						
Coronary atherosclerosis and other heart disease	0.00	10.22	0.94	0.78	49.73	10.68
Menstrual disorders	10.57	2.33	1.40	26.36	2.13	19.18
Residual codes; unclassified	0.83	3.10	0.00	13.44	26.33	19.18
Osteoarthritis	0.24	6.21	0.31	2.33	33.24	14.79
Screening and history of mental health and substance abuse codes	5.46	8.15	0.94	1.81	20.74	9.86

Characteristics of utilities, demographics and chronic disease conditions were summarized for each cluster. All the data shown within the headers of demographics and chronic disease diagnosis were expressed in percentages (%).

To test the hypothesis that ED revisit high-risk patients can be partitioned into subgroups with similar patterns of demographics, primary diagnosis and procedure, and chronic disease conditions to allow future targeted care, high risk patients were clustered with unsupervised analysis. Our prospective analysis (Figure 7, right panel) revealed a pattern of six distinct sub-groups among the high-risk patients, and these clinically relevant clusters (Table 1) grouped around multiple “anchoring” demographic and chronic disease conditions with different ED resource utilization patterns. The largest cluster (#1) was characterized by over 93.7% young adult patients (between the ages of 18 and 35). Cluster #1 also featured the lowest average ED counts and lowest cost consumption in future 30 days. Cluster #4 had patients in the similar age group as cluster #1 (98.5% in age 18–35), but most of them were female (89.7%) and had asthma diagnosis (74.4%). In contrast, cluster #5 contained a relatively senior (61.4% in age>50 age group) population with highest future 30-day cost, and the highest average consumption of laboratory and radiology tests in the post ED discharge 30 days. Cluster #6 and #5 shared similar age patterns (Cluster #6, 42.4% in the age>50 group). However, cluster #6 was mainly composed of female (81.1% of female), while cluster #5 had more male patients (61.7% of male). Encounters in cluster #5 generally had higher percentages of chronic disease diagnoses than cluster #6, with exclusion of asthma, headache and menstrual disorders. Clusters #2 and 3 had similar sex groups where most were males (70.1% in cluster #2 and 79.7% in cluster #3). The health status of the two clusters were different, however. 71.0% of the encounters in cluster #3 had no chronic disease while all the encounters in cluster #2 had chronic diseases. Cluster #2 had the highest total future 30-day cost among the all six clusters.

**Figure 8:**
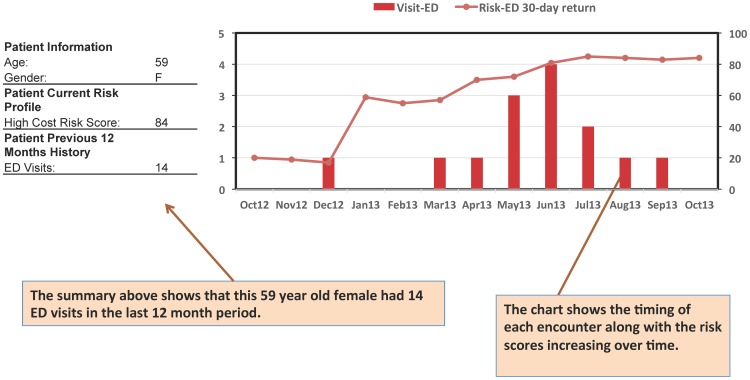
A prospective case study on monthly ED visits and risks for a patient.

A prospective case-study chart, for a patient randomly selected from the prospective cohort, was shown in Figure 8. As the risk score changed longitudinally from low risk (<20) to high risk (>80), the corresponding ED 30-day visit count increased accordingly from 0 to a peak value of 4. The correlation between the 12-month profile of the ED visits and risk score indicated the utilities of our predictive model.

## Discussion

We developed an ED revisit risk model estimating patients' ED 30-day revisit risks, ranging from 1 to 100. Retrospective and prospective testing results as well as a case study summary demonstrated our algorithm's effectiveness in the identification of patients with different ED revisit risks with decent sensitivities. Particularly the sensitivity reached 24.4% for encounters with 30–40 risk scores, which was much higher than the best result to our knowledge that reported an 8% sensitivity among high risk samples for 12-month ED revisit rate prediction [47].

We implemented a prospective utilization interface integrating the predictive algorithm with a visualization dashboard, allowing age-group filters to examine prospectively the model performance in different age sub cohorts. The PPV and sensitivity above a risk score of 80 were 75.6% and 2.9% for patients at 13–18 age group, 81.6% and 11.2 for patients at 19–34 age group, 85.4% and 13.7% for patients at 35–49 age group, 83.9% and 10.2% for patients at 50–65 age group, and 76% and 2.6% for patients above 65 age group. In addition, pediatric patents are unique in clinical research and need special attention as a future direction of our predictive analytics.

We have marshaled the Maine HIE EMR records, through necessary rigorous mapping of multiple providers' data to standard nomenclature including LOINC [48], RXNorm [49], and SNOMED [50], and developed our enterprise data warehouse (EDW). This warehouse offers an un-paralleled data repository that can be leveraged to realize value through the application of advanced analytic techniques. Applying analytical tools on EMR and HIE data, including our ED model and the high-risk patient clustering method, will help health care providers effectively leverage their EMR to better understand ED service delivery while providing opportunities for improved healthcare delivery for the patients. However, HIE has its own limitations. It doesn't include the mental health and substance abuse diagnostic information as it is in compliance with Maine state law that prevents the reporting of these codes to HIN. These kinds of conditions however were shown to be frequent within the ED patient population [51]. According to a national health statistic report of US in 2005, less than 5% of hospital admissions were due to mental disorders, in which around 2.7% had ED revisits within 7 days [52]. A study of hospitalization in Washington State in 2007 revealed that 4.6% of hospital visits had mental health-related diseases with 62% having ED admissions [53]. Another investigation of ED patients in one state reported 57% of patients with mental disorder diagnosis had multiple ED visits in one year [54]. An analysis of national Medicaid data of 2005 demonstrated that 9.7% of self-arm patients had ED returns in 30 days' period [51]. We applied our current algorithm prospectively on the sub-cohort with missing diagnosis codes, and found that there were still a reasonable number of encounters with ED 30-day return identified by our model: there were 15,160 encounters with missing diagnosis codes having ED 30-day revisits in the prospective cohort, in which 2396 encounters were high risk (risk score>70) reaching a sensitivity of 15.8%. It was partly because other available clinical information such as outpatient prescription information of those encounters helps maintain the model performance. In addition to the mental disease diagnostic codes, self-rated health conditions, life style related factors, and socioeconomic status are not currently available for our predictive analytics. Population with missing diagnoses information will be modeled separately with appropriate diagnostic codes including mental disorder codes, once the HIE is applied to hospitals where the mental health and substance abuse diagnostic data can be released. Other missing information will also be added to the database for our model improvement. Therefore, we expect our current model can be significantly improved with more comprehensive information. Furthermore, while HIE data represents an ideal source of community-wide/regional patient data, operational HIEs are not present in all States. Although the samples collected from HIE for our study were with all ages, all payers and all diseases in Maine State, they may have unexpected bias and not exactly match the nationwide population characteristics and ED visit trends. After overcoming these limitations, our ED predictive model will be improved with a broader applicability in health care globally.

Variance analysis and two rounds of decision tree modeling process were carried out sequentially for feature selection. 127 out of 14,680 features were chosen for the final ensemble model development. Sensitivity was plotted as a function of feature numbers in Figure S3. To achieve optimal learning and avoid under or over fitting, 127 features were selected. Comparatively, we performed LACE index analysis, including length of stay, ED visit history, and comorbidities with 14 types of conditions. The LACE index performance however was poor with c-statistics of 0.57 in both retrospective and prospective analysis. We also compared our model with a simple model using age as the only discriminant feature. The c-statistics of the latter was only 0.527, showing a low predictive power. These comparisons support the competitive performance of our model in regard to predict ED 30-day revisit. The interface among the electronic medical record (EMR), hand-held devices (cell phone), and cloud-based clinical-computation may benefit clinicians and remove the entry barrier of a sophisticated model.

Reasons of ED revisits were analyzed using our model. Between January 1 and June 30, 2013, nearly 5000 ED returned 30 days post discharge with the same diagnoses and/or procedures as their initial visits, partly indicating inappropriate care they received at the first time. Unlike revisits due to unrelated causes to the initial visit, revisits caused by the same reasons are usually avoidable by targeted intervention. Either more definitive diagnoses or refined discharge plans can help to prevent this sub cohort from revisiting. Plenty of ED revisits were unnecessary as a result of overestimation or medical errors. By comparing the predicted and the real ED revisits, the redundant resource usage can be estimated, leading to a measure of the healthcare quality. Further characterization of those redundant ED visits will help the care providers to understand the causes of the unnecessary ED expense and thereby to approach a more cost-effective usage plan in the future.

Learning the unique patterns of the patients with high risk of reusing the medical service is another application of our method. We sought to determine whether those patterns existed among the considerable heterogeneity of the high-risk patient population when considered together. Our unsupervised clustering analysis revealed six clinically relevant subgroups among the high-risk patient population that were confirmed as durable upon prospective testing. These subgroups had unique patterns of demographics, disease severities, comorbidities and resource consumption. This finding revealed a new opportunity for targeted and proactive intervention to prevent ED revisit. For example, cluster #5 and #6 both represented 0.2% of the entire prospective cohort consuming 25.3% (cluster #5) and 14.6% (cluster #6) of all ED revisit high-risk group resource utilization (total medical expense), which agreed with the findings from other studies that there were few percentage of people consuming relatively high resource [55], [56], suggesting a new care management strategy to focusing on these patients for an effective cost reduction. We noted a decreased prevalence of the co-occurring chronic conditions in four other cluster groups of relatively younger adults with much less resource consumption. 29.0% of cluster #3 subjects, who were not associated with any chronic disease history, may benefit from targeted care management to keep them out of the emergency room. Currently, many existing care management strategies are directed toward single conditions. Our clustering results, however, demonstrated that ED resource utilization is driven by a variety of demographic and clinical factors. Therefore, with our ED risk stratification analytics, we propose new strategies of coordinated care, which we speculate may lead to greater case management efficacy.

We believe that the use of this model will benefit both healthcare providers and patients. With our prospective-validated ED risk model, health care providers can reasonably estimate the ED revisit risks at the patient discharge time. Such pre-knowledge will provide a perspective of health care economics for the future clinical resource related to ED. Given various health care services are currently integrated to each other, with our ED predictive analytics, healthcare resources distributing among the inpatient, outpatient, ED and others could be balanced and re-allocated in advance with consideration of the forecasted future ED reuse. In this regard, the identification of the high-risk group can lead to targeted care with better patient experience, and effective resource utilization. In addition, as an early warning tool, the predicted ED revisit risk profiles can raise patients' self-awareness to achieve better self-management. Therefore, the integration of our ED risk tool can definitely improve care quality and drive the reduction of the unnecessary ED revisits.

## Supporting Information

Figure S1 **“Time to event” analysis for retrospective patients with 30 day ED revisits post ED discharge.** Percentage of the patients who didn't return to ED in a time frame from 0 to 30 days post ED discharge.
(TIFF)

Figure S2 **Feature selection process.** A flow chart showing the procedures to reduce the 14,680 features to 127 features before training the model.
(TIFF)

Figure S3 **Sensitivity of the predictive model versus the selected feature number.** A curve showing the identified rates of ED 30-day return event, using the predictive models that were built by different feature numbers.
(TIFF)

Table S1 **EMR features used to develop the model.** A list of EMR features that used as the predictors for the model training.
(DOCX)

Table S2 **Patient characteristics.** A summary of patient characteristics in the retrospective and prospective cohorts.
(DOCX)

Table S3 **ED 30 days revisit risk stratification results of all encounters: retrospective and prospective.** The model performances within different risk score ranges between 0 and 100, in retrospective and prospective cohorts.
(DOCX)
