# Correction: Risk Prediction of Emergency Department Revisit 30 Days Post Discharge: A Prospective Study

**DOI:** 10.1371/journal.pone.0117633

**Published:** 2015-01-28

**Authors:** The PLOS ONE Staff

The affiliation for the first author is incorrect. Shiying Hao is not affiliated with #1. His only affiliation is #2 Department of Surgery, Stanford University, Stanford, California, United States of America.
