# Supplementary figures and images for: Risk Prediction of Emergency Department Revisit 30 Days Post Discharge: A Prospective Study
Source: PLoS One. 2014 Nov 13;9(11):e112944. doi: 10.1371/journal.pone.0112944 (PMC4231082; doi:10.1371/journal.pone.0112944)

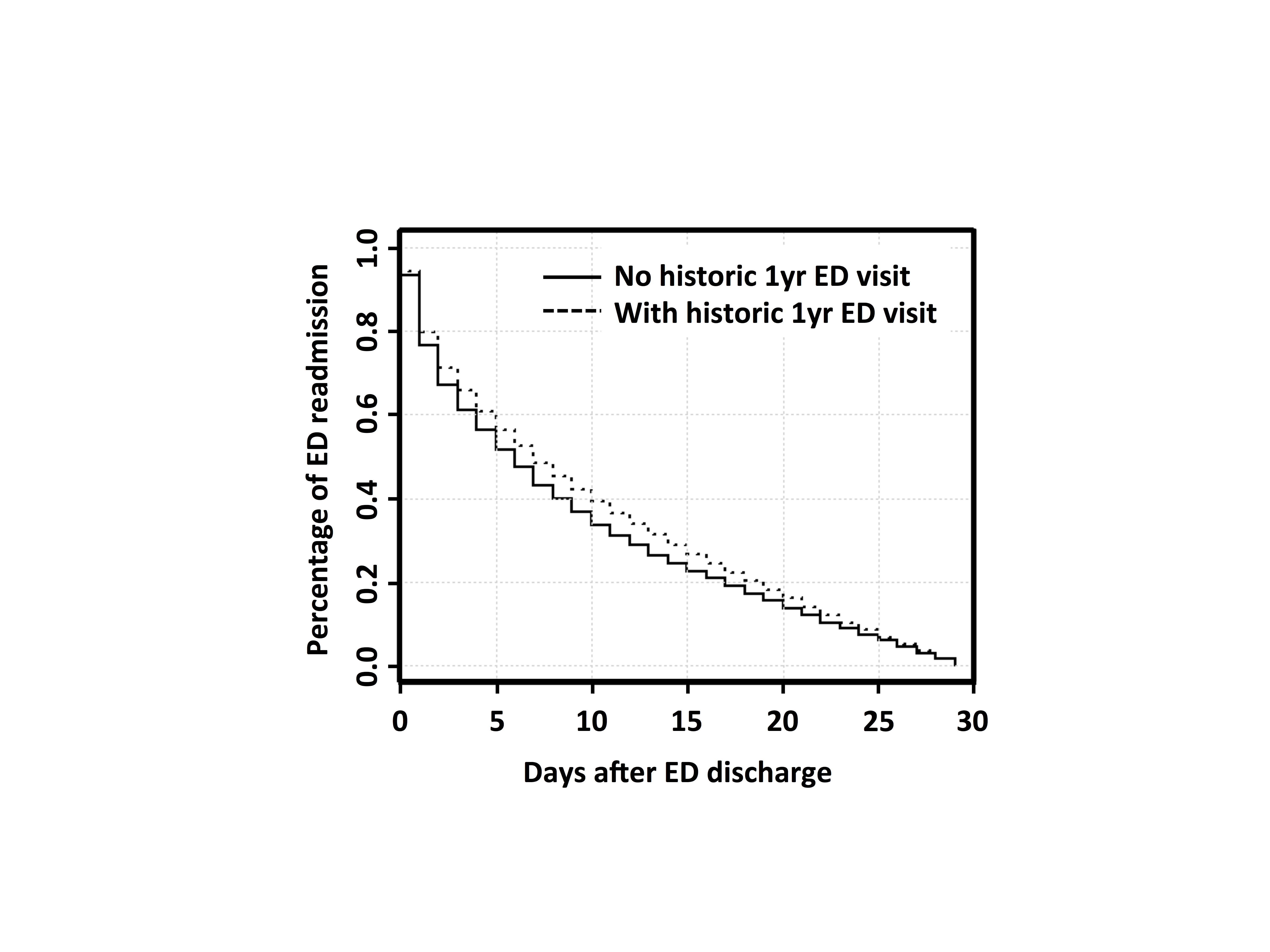

Supplement: Figure S1 — “Time to event” analysis for retrospective patients with 30 day ED revisits post ED discharge. Percentage of the patients who didn't return to ED in a time frame from 0 to 30 days post ED discharge. (TIFF) [file pone.0112944.s001.tiff]

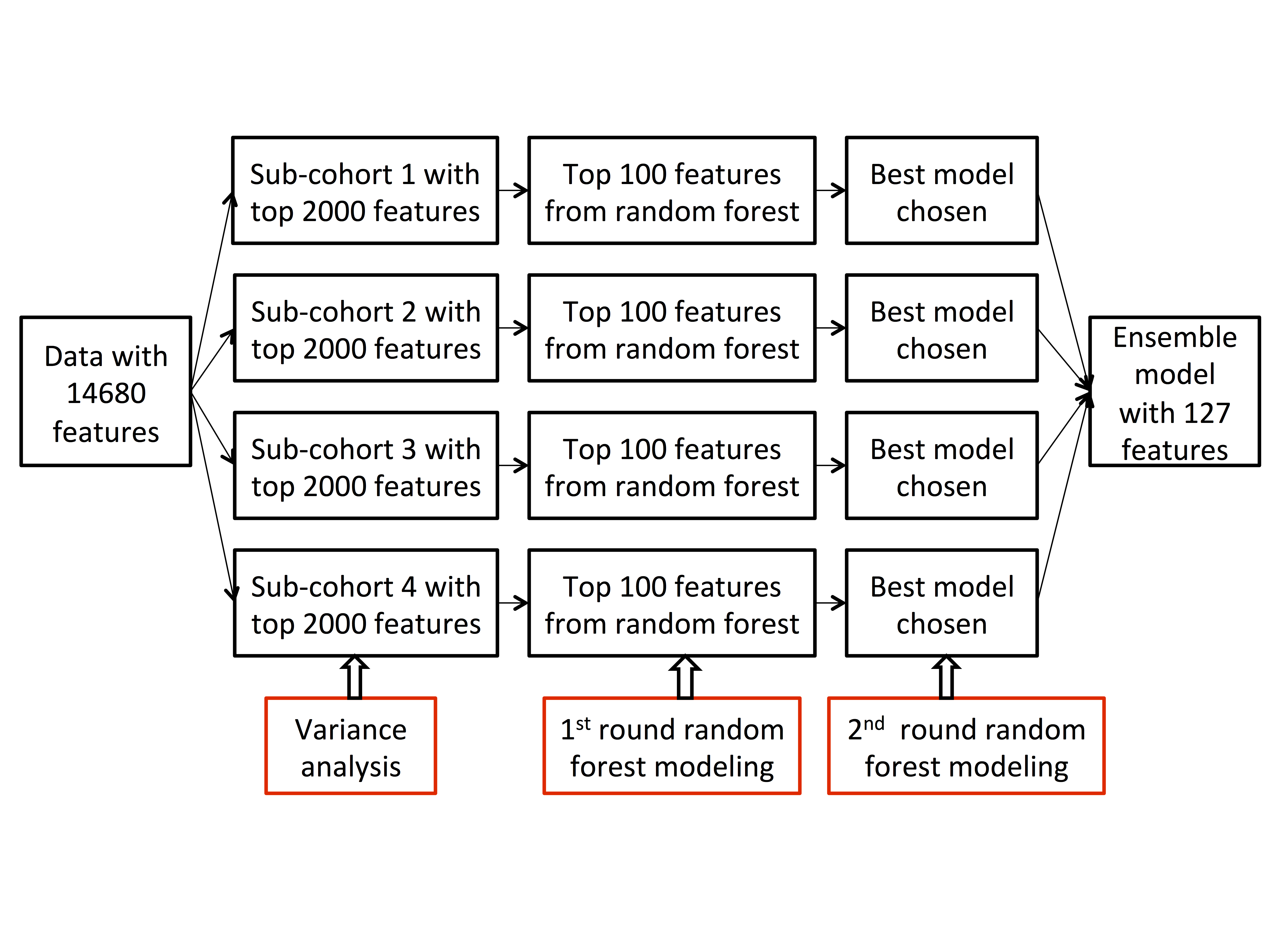

Supplement: Figure S2 — Feature selection process. A flow chart showing the procedures to reduce the 14,680 features to 127 features before training the model. (TIFF) [file pone.0112944.s002.tiff]

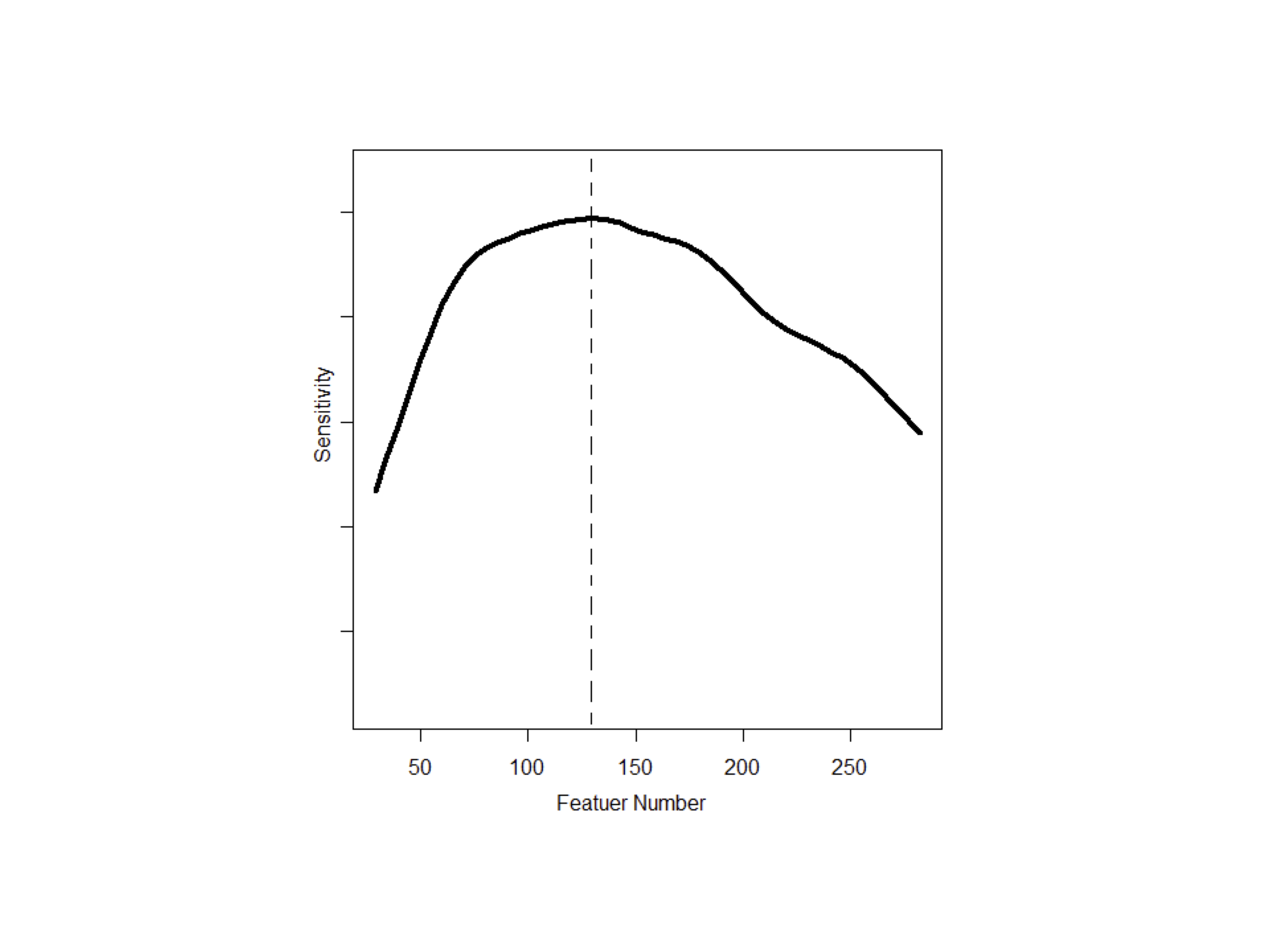

Supplement: Figure S3 — Sensitivity of the predictive model versus the selected feature number. A curve showing the identified rates of ED 30-day return event, using the predictive models that were built by different feature numbers. (TIFF) [file pone.0112944.s003.tiff]
