# Supplementary material for: Risk Prediction of Emergency Department Revisit 30 Days Post Discharge: A Prospective Study
Source: PLoS One. 2014 Nov 13;9(11):e112944. doi: 10.1371/journal.pone.0112944 (PMC4231082; doi:10.1371/journal.pone.0112944)
Supplement: Table S1 — EMR features used to develop the model. A list of EMR features that used as the predictors for the model training. (DOCX) [file pone.0112944.s004.docx]

| Table S1. EMR features used to develop the model | | |
| --- | --- | --- |
| Feature group | Feature number | Feature description (12 month clinical history before ED discharge) |
| Encounter history | 84 | Visit counts of different encounter types (E/O/I/P/R) * |
|  |  | The accumulated length of hospitalized stay |
|  |  | Counts of historical chronic disease diagnoses |
|  |  | Counts of total and no redundant total radiographic and laboratory tests, and outpatient prescriptions |
| Demographics |  | Female, male |
|  | 9 | Income, education, payer |
|  |  | Age group is defined by age at ED admission |
|  |  | (0, 1-5yr, 6-12yr, 13-18yr, 19-34yr, 35-49yr, 50-65yr, 65+yr) ** |
| Facility | 10 | Different facilities |
| Procedure | 1 | Counts for different primary procedure and secondary procedure |
| Chronic disease condition | 8 | Counts for chronic disease diseases |
| Diagnosis | 8 | Counts for primary diagnosis and secondary diagnosis |
| Laboratory test | 2 | Counts for different laboratory test results |
| Outpatient prescriptions | 5 | Counts for different outpatient prescriptions |
| * Encounter type descriptions: E-Emergency, O-Outpatient, I-Inpatient, P-Pre admission, R-Recurring admission, **yr-year | | |
