# Supplementary material for: Risk Prediction of Emergency Department Revisit 30 Days Post Discharge: A Prospective Study
Source: PLoS One. 2014 Nov 13;9(11):e112944. doi: 10.1371/journal.pone.0112944 (PMC4231082; doi:10.1371/journal.pone.0112944)
Supplement: Table S2 — Patient characteristics. A summary of patient characteristics in the retrospective and prospective cohorts. (DOCX) [file pone.0112944.s005.docx]

| Table S2. Patient characteristics | | | | |
| --- | --- | --- | --- | --- |
|  | Retrospective | | Prospective | |
|  | (Jan.1, 2012 – Dec.31, 2012) | | (Jan.1, 2013 – Jun.30, 2013) | |
|  | Control | Case | Control | Case |
|  | N = 236599 | N = 56862 | N = 154045 | N = 39841 |
| Gender | | | | |
| Female | 53.43% | 53.66% | 53.58% | 52.95% |
| Male | 46.57% | 46.34% | 46.42% | 47.05% |
| Age | | | | |
| Median(IQR) | 36.83 | 38.04 | 37.16 | 38.90 |
|  | (20.81, 57.26) | (24.78, 56.54) | (21.15, 57.51) | (25.31, 56.26) |
| Median family income estimate | | | | |
| Median(IQR) | 58745 | 55108 | 58116 | 55108 |
|  | (48810, 63750) | (47644, 63030) | (47644, 68000) | (47644, 63440) |
| Percent high school graduate or higher | | | | |
| Median(IQR) | 89.40 | 88.90 | 89.40 | 88.70 |
|  | (86.40, 92.40) | (86.00, 91.40) | (86.40, 92.60) | (86.00, 91.80) |
| Percent bachelor's degree or higher | | | | |
| Median(IQR) | 22.20 | 21.70 | 22.20 | 22.00 |
|  | (16.60, 28.30) | (15.00, 27.00) | (15.20, 30.50) | (14.60, 28.70) |
