# Supplementary material for: Risk Prediction of Emergency Department Revisit 30 Days Post Discharge: A Prospective Study
Source: PLoS One. 2014 Nov 13;9(11):e112944. doi: 10.1371/journal.pone.0112944 (PMC4231082; doi:10.1371/journal.pone.0112944)
Supplement: Table S3 — ED 30 days revisit risk stratification results of all encounters: retrospective and prospective. The model performances within different risk score ranges between 0 and 100, in retrospective and prospective cohorts. (DOCX) [file pone.0112944.s006.docx]

| Table S3. ED 30 days revisit risk stratification results of all encounters: retrospective and prospective | | | | | | | | | | |
| --- | --- | --- | --- | --- | --- | --- | --- | --- | --- | --- |
| Retrospective (Jan. 1, 2012 - Dec. 31, 2012) | | | | | | | | | | |
| Characteristic | Risk score range | | | | | | | | | |
|  | 0-10 | 10-20 | 20-30 | 30-40 | 40-50 | 50-60 | 60-70 | 70-80 | 80-90 | 90-100 |
| No. of ED encounters | 27755 | 16424 | 8505 | 23454 | 8276 | 6205 | 5219 | 1080 | 779 | 239 |
| Positive predictive value | 0.088 | 0.129 | 0.150 | 0.191 | 0.260 | 0.360 | 0.553 | 0.539 | 0.770 | 0.891 |
| Sensitivity | 0.128 | 0.112 | 0.067 | 0.236 | 0.113 | 0.118 | 0.152 | 0.031 | 0.032 | 0.011 |
| Specificity | 0.679 | 0.819 | 0.908 | 0.760 | 0.922 | 0.950 | 0.970 | 0.994 | 0.998 | 1.000 |
| Average ED visits in the future 30 days | 0.351 | 0.447 | 0.533 | 0.597 | 0.950 | 1.301 | 1.756 | 2.103 | 2.899 | 4.201 |
| Prospective (Jan. 1, 2013 – Jun. 30, 2013) | | | | | | | | | | |
| Characteristic | Risk score range | | | | | | | | | |
|  | 0-10 | 10-20 | 20-30 | 30-40 | 40-50 | 50-60 | 60-70 | 70-80 | 80-90 | 90-100 |
| No. of ED encounters | 52363 | 34312 | 19088 | 47430 | 16791 | 11657 | 8861 | 1888 | 1296 | 200 |
| Positive predictive value | 0.098 | 0.132 | 0.163 | 0.205 | 0.290 | 0.392 | 0.605 | 0.643 | 0.858 | 0.935 |
| Sensitivity | 0.129 | 0.114 | 0.078 | 0.244 | 0.122 | 0.115 | 0.135 | 0.030 | 0.028 | 0.005 |
| Specificity | 0.693 | 0.807 | 0.896 | 0.755 | 0.923 | 0.954 | 0.977 | 0.996 | 0.999 | 1.000 |
| Average ED visits in the future 30 days | 0.372 | 0.464 | 0.559 | 0.633 | 1.031 | 1.437 | 2.103 | 2.816 | 3.883 | 4.490 |
